# The Effect of Diabetes-Specific Enteral Nutrition Formula on Cardiometabolic Parameters in Patients with Type 2 Diabetes: A Systematic Review and Meta–Analysis of Randomised Controlled Trials

**DOI:** 10.3390/nu11081905

**Published:** 2019-08-15

**Authors:** Omorogieva Ojo, Sharon Marie Weldon, Trevor Thompson, Rachel Crockett, Xiao-Hua Wang

**Affiliations:** 1 Department of Adult Nursing and Paramedic Science, University of Greenwich, London SE9 2UG, UK; 2 Barts Health NHS Trust, The Royal London Hospital, Whitechapel Rd, Whitechapel E1 1BB, UK; 3 Department of Psychology, University of Greenwich, London SE10 9LS, UK; 4 Division of Psychology, Faculty of Natural Sciences, University of Stirling, Scotland FK9 4LA, UK; 5 The School of Nursing, Soochow University, Suzhou 215006, China

**Keywords:** diabetes specific formula, standard formula, type 2 diabetes, enteral nutrition, enteral tube feeding, lipids, fasting blood glucose, glycated haemoglobin

## Abstract

Background: The prevalence of diabetes is on the increase in the UK and worldwide, partly due to unhealthy lifestyles, including poor dietary regimes. Patients with diabetes and other co-morbidities such as stroke, which may affect swallowing ability and lead to malnutrition, could benefit from enteral nutrition, including the standard formula (SF) and diabetes-specific formulas (DSF). However, enteral nutrition presents its challenges due to its effect on glycaemic control and lipid profile. Aim: The aim of this review was to evaluate the effectiveness of diabetes-specific enteral nutrition formula versus SF in managing cardiometabolic parameters in patients with type 2 diabetes. Method: This review was conducted in accordance with the preferred reporting items for systematic reviews and meta-analyses. Three databases (Pubmed, EMBASE, PSYCInfo) and Google scholar were searched for relevant articles from inception to 2 January 2019 based on Population, Intervention, Comparator, Outcomes and Study designs (PICOS) framework. Key words, Medical Subject Heading (MeSH) terms, and Boolean operators (AND/OR) formed part of the search strategy. Articles were evaluated for quality and risks of bias. Results: Fourteen articles were included in the systematic review and five articles were selected for the meta-analysis. Based on the findings of the review and meta-analysis, two distinct areas were evident: the effect of DSF on blood glucose parameters and the effect of DSF on lipid profile. All fourteen studies included in the systematic review showed that DSF was effective in lowering blood glucose parameters in patients with type 2 diabetes compared with SF. The results of the meta-analysis confirmed the findings of the systematic review with respect to the fasting blood glucose, which was significantly lower (*p* = 0.01) in the DSF group compared to SF, with a mean difference of −1.15 (95% CI −2.07, −0.23) and glycated haemoglobin, which was significantly lower (*p* = 0.005) in the DSF group compared to the SF group following meta-analysis and sensitivity analysis. However, in relation to the sensitivity analysis for the fasting blood glucose, differences were not significant between the two groups when some of the studies were removed. Based on the systematic review, the outcomes of the studies selected to evaluate the effect of DSF on lipid profile were variable. Following the meta-analysis, no significant differences (*p* > 0.05) were found between the DSF and SF groups with respect to total cholesterol, LDL cholesterol and triglyceride. The level of the HDL cholesterol was significantly higher (*p* = 0.04) in the DSF group compared to the SF group after the intervention, with a mean difference of 0.09 (95% CI, 0.00, 0.18), although this was not consistent based on the sensitivity analysis. The presence of low glycaemic index (GI) carbohydrate, the lower amount of carbohydrate and the higher protein, the presence of mono-unsaturated fatty acids and the different amounts and types of fibre in the DSF compared with SF may be responsible for the observed differences in cardiometabolic parameters in both groups. Conclusion: The results provide evidence to suggest that DSF is effective in controlling fasting blood glucose and glycated haemoglobin and in increasing HDL cholesterol, but has no significant effect on other lipid parameters. However, our confidence in these findings would be increased by additional data from further studies.

## 1. Introduction

Diabetes is a metabolic condition which is characterised by chronic hyperglycaemia and is caused by a range of factors including genetic inheritance and environmental influences [1]. The prevalence of diabetes is on the increase in the UK and worldwide, partly due to the changes in lifestyle, including lack of physical activity and unhealthy diets, which lead to overweight and obesity [2,3,4,5]. In addition, improvements in technology and the greater awareness of the condition have meant that diabetes is now better detected and more people are engaging in screening programmes. About 90% of patients with diabetes are diagnosed with type 2 diabetes [6,7,8]. The impact of diabetes on the people living with the condition can be profound in terms of morbidity and mortality, as well as a cost burden to the National Health Service (NHS). Individuals with diabetes are more likely to be admitted to hospital and it can have a significant effect on the quality of life of patients [9,10]. Diabetes is a major risk factor for kidney dysfunction, lower limb amputations, retinopathy, cardiovascular disease and other co-morbidities such as stroke, which can lead to swallowing problems and malnutrition. Based on these issues, diabetes continues to be a major public health concern in the UK and globally, and strategies for managing the condition continue to evolve. Often, management relies on lifestyle modifications such as increased physical activity levels and the use of dietary interventions in order to prevent the onset of type 2 diabetes and ultimately reduce the possibility of diabetic complications [11]. However, in patients who are sedentary and immobile, the use of physical activity as a strategy for managing the condition is sometimes impracticable.

Therefore, individuals with diabetes and other conditions, such as stroke, which could affect mobility and swallowing ability, may benefit from enteral nutrition such as oral nutrition supplements and the use of a nasogastric feeding tube (for short-term feeding) or a percutaneous endoscopic gastrostomy tube for long-term intervention to deliver enteral feeds and formulas [12]. Usually, these individuals have functional guts and the essence is to provide adequate nutrition, hydration and medication to these patients in order to improve their nutritional status and clinical outcomes, including quality of life.

Why it is important to do this review:

The current review focuses mainly on patients with type 2 diabetes from a range of backgrounds, including those attending diabetes centre/outpatient diabetic clinics, rehabilitation departments, ambulatory patients, nursing homes and long-term care facilities, and intensive care units. Patients with diabetes are at a greater risk of developing stroke, peripheral vascular disease, renal impairment and dementia compared with those without the condition due to chronic hyperglycaemia [13]. The long-term complications of diabetes, including its co-morbidities, have implications for the length of hospital stay. Thus, while the average length of hospital stay in patients with diabetes as the primary diagnosis has been estimated to be 4.3 days, it is 8 days in patients with additional diagnoses and 3.1 days in all hospitalisations [14]. The use of enteral feeding in patients with diabetes can present a range of challenges in the control of blood glucose levels and other cardiometabolic parameters [15]. These parameters, including lipid profile, such as total cholesterol, high density lipoprotein cholesterol, low density lipoprotein cholesterol and triglyceride, are important biomarkers in patients with type 2 diabetes as they have implications for insulin resistance and cardiovascular mortality.

In patients with diabetes who are on enteral nutrition, the enteral feeds provided can be in the form of either Standard Formulas (SF) or Diabetes Specific Formulas (DSF). Enteral feeding formulas have a tendency to promote hyperglycaemia and insulinemic responses in patients with diabetes and in healthy subjects [16,17]. In addition, the effect of enteral nutrition on blood glucose parameters may be due to the fact that continuous enteral feeding is a source of continuous supply of glucose, providing 10–20 g of carbohydrates per hour, which is not the same during normal eating [15]. The absence of the normal postprandial glucose peak in patients with diabetes on enteral nutrition makes the management of hyperglycaemia difficult [15]. On the other hand, the effect of different types and amounts of fibre and mono-unsaturated fatty acids in various enteral feeds may influence lipid profile and other cardiometabolic parameters such as fasting blood glucose and glycated haemoglobin in patients with type 2 diabetes [18]. The role of the different enteral feeding formulas such as SF and DSF and their impact on cardiometabolic parameters in patients with diabetes continues to generate interest and controversy, and there appears to be no consensus among researchers on the most effective management strategy for these patients.

DSFs usually contain carbohydrates with low GI such as fructose and large amounts of monounsaturated fatty acids in varying amounts, which have effect on glycaemic control [17,18,19,20]. On the other hand, SFs are often high in carbohydrate and contain only low to moderate levels of lipids and do not have dietetic fibre [17].

Previous reviews on the use of enteral nutrition in patients with diabetes [16,17,21,22,23] either lacked consensus in the recommendations, were based only on glycaemic control or did not involve meta-analysis. In addition, concerns remain with the use of DSF in terms of the safety and tolerance of relatively high levels of fat and fructose with respect to lipid metabolism and lactic acidosis, despite its advantage in improving blood glucose compared with SFs [16,19]. Therefore, this review provides a quantitative assessment of the relative effectiveness of DSF compared with SF.

Aim: The aim was to evaluate the effectiveness of diabetes specific enteral nutrition formula versus SF in managing cardiometabolic parameters in patients with type 2 diabetes.

## 2. Methods

This study was conducted in accordance with the preferred reporting items for systematic reviews and meta-analyses (PRISMA) [24].

### 2.1. Types of Studies and Participants

Only randomised controlled studies were included in this review and participants were patients with type 2 diabetes.

Inclusion and Exclusion Criteria.

The criteria for considering studies for the review are outlined in Table 1.

### 2.2. Type of Intervention

The intervention for this review was based on diabetes-specific enteral formula, irrespective of the type of feeding tube, mode and rate of delivery of the enteral feed and clinical settings.

### 2.3. Types of Outcome Measures

The following were the outcome measures of interest;

- Blood glucose parameters—Fasting blood glucose and glycated haemoglobin.
- Lipid profile: Total cholesterol, low density lipoprotein (LDL) cholesterol, high density lipoprotein (HDL) cholesterol and triglycerides.

### 2.4. Search Strategy

Databases encompassing Pubmed, EMBASE, PSYCInfo and Google scholar were searched for relevant articles based on the Population (Patients with diabetes), Intervention (Diabetes Specific Formula), Comparator (Standard enteral formulas), Outcomes (outcome measures) and Study designs (Randomised controlled studies)—PICOS framework (Table 2) [25]. The use of key words, truncation symbols, Medical Subject Heading (MeSH) terms and Boolean operators (AND/OR) formed part of the search strategy. Searches were conducted from the date of inception of databases until 2 January 2019.

**Figure 1:**
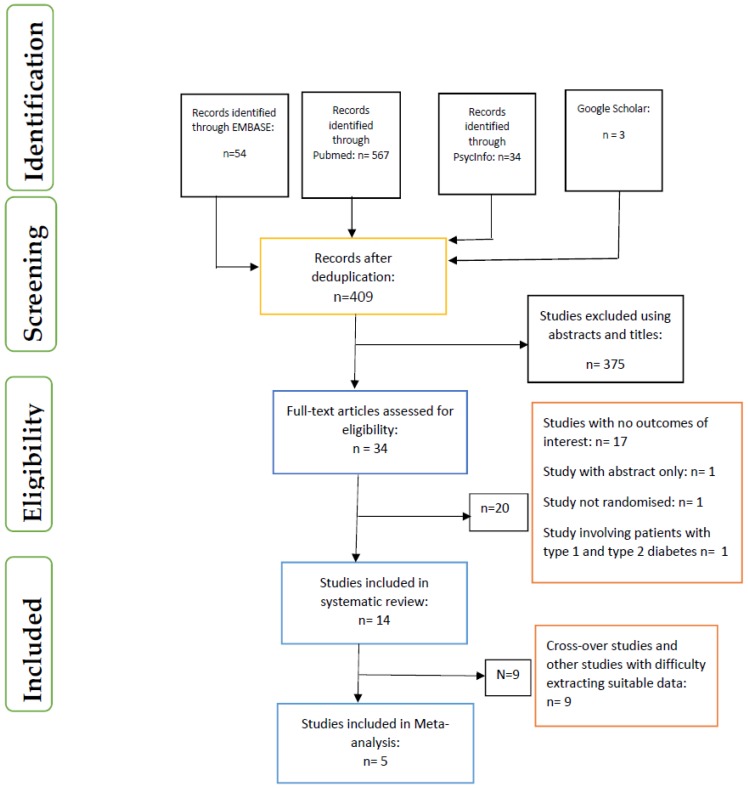
PRISMA flow chart on selection and inclusion of studies.

The screening of studies and the evaluation of their eligibility and inclusion were in line with PRISMA [24] guidelines (Figure 1). These procedures were conducted by five researchers (OO, SMW, TT, RC, X-HW) and differences were resolved through consensus.

### 2.5. Data Extraction

All the articles from different databases were exported to ENDNote (Analytics, Philadelphia, PA, USA) for de-duplication. Data extraction was carried out by one researcher (OO) and cross-checked by the other four researchers (SMW, TT, RC, X-HW).

### 2.6. Assessment of Risk of Bias and Evaluation of Quality

A critical appraisal skills programme (CASP) tool was used to appraise the quality of the articles [26]. In addition, the researchers carried out an assessment of the risk of bias using the domain-based tool (random sequence generation, allocation concealment, blinding of participants, personnel and outcome assessment, reporting bias and selective reporting) to evaluate the studies included [27].

### 2.7. Statistical Analysis

Articles that met the inclusion criteria for meta-analysis were exported to RevMan (Review Manager, 5.3) [28] for data analysis. Therefore, cross-over studies and other studies which presented with difficulty in extracting suitable data were excluded from the meta-analysis. The data analysis included both meta-analysis and sensitivity analysis, the latter being conducted to test the consistency of the effect of DSFs on the different cardiometabolic paramters. The random effects model was used for the parameters of interest due to the high level of heterogeneity measured by the statistic *I*^2^ with values ranging from 34% to 100%. A p value of 0.10 was used to determine the statistical significance of heterogeneity.

### 2.8. Effect Size

A forest plot was used to present the results of the meta-analysis and statistical significance for the overall effect of the intervention was determined by a *p* value of <0.05.

## 3. Results

### 3.1. Data Inclusion Decisions

Fasting blood glucose in the studies included was measured after overnight fasting, using standard measuring instruments. This is the standard method of measuring fasting blood glucose: the blood glucose concentrations were expressed as Means. However, the studies by Pohl et al. [29,30] were expressed as median and interquartile ranges and these were converted to means and standard deviations [27]. Fourteen studies were included in the systematic review (Table 3) while only five studies [29,30,31,32,33] were selected for the meta-analysis (Table 4).

### 3.2. Assessment of Risk of Bias in Included Studies

**Figure 2:**
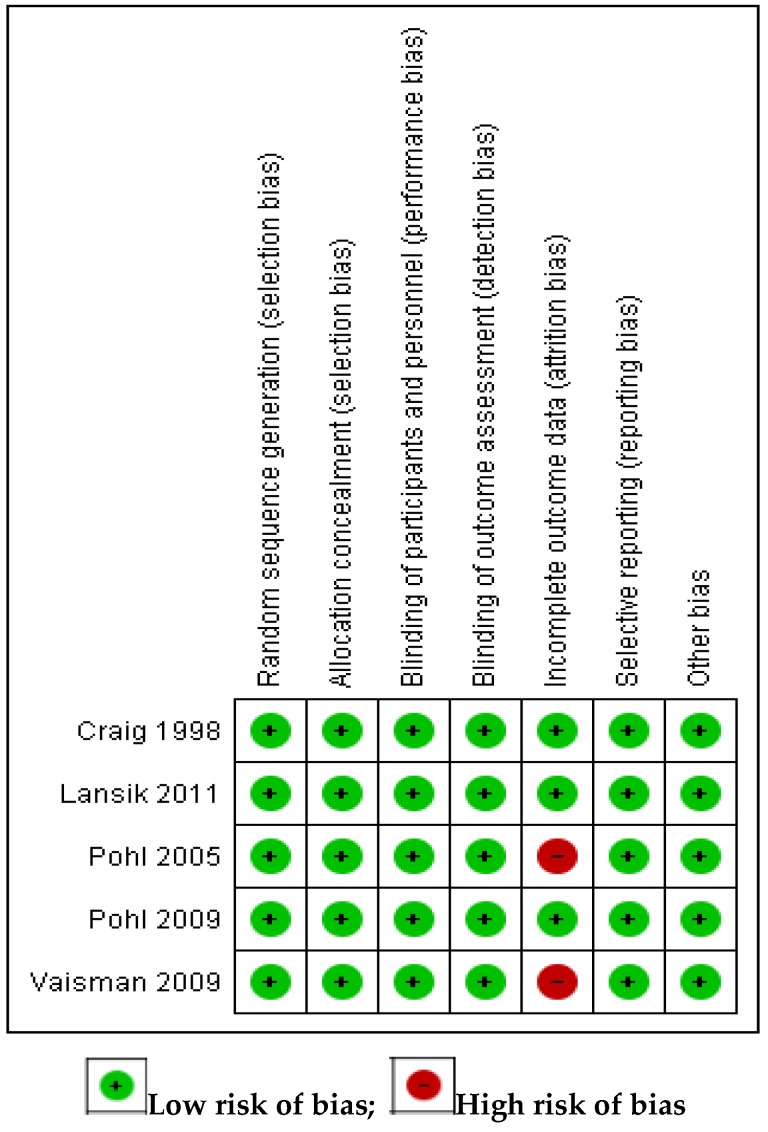
Risk of bias summary.

Figure 2 shows the risk of bias summary of the various studies included in the meta-analysis. All the studies demonstrated low risk of bias in all the areas, except with respect to incomplete outcome data (attrition bias) where two studies [29,33] showed high risk of bias.

Based on the findings of the review and the meta-analysis, two distinct areas were evident: the effect of DSF on blood glucose parameters and the effect of DSF on lipid profile.

The effect DSF on blood glucose parameters:

All the fourteen studies included in the systematic review showed that DSF was effective in lowering blood glucose parameters in patients with type 2 diabetes compared with SF. In particular, DSF improved glycaemic control and lowered insulin requirements [18,29,31,35,36,37,38,39]. It provided better clinical outcomes, including reducing the risk of acquired infections and pressure ulcer, reduced body weight and was safer compared to SF [29,31,32]. In addition, the use of DSF was shown to be effective in lowering postprandial blood glucose levels compared to SF [20,32,34,40].

**Figure 3:**
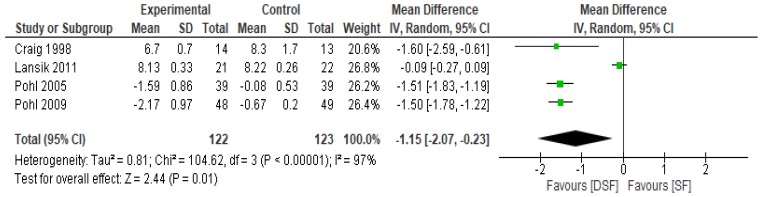
The effect of DSF on fasting blood glucose (mmol/L).

Pohl et al. [30] observed that long-term tube feeding with a DSF significantly lowered fasting blood glucose and improved glycaemic control. Similarly, Vaisman et al. [33] reported that DSF significantly improved longer-term glycaemic control in diabetic patients compared to SF. The results of the meta-analysis confirmed the findings of the systematic review. With respect to the fasting blood glucose, it was significantly lower (*p* = 0.01) in the DSF group compared to SF, with a mean difference of −1.15 (95% CI −2.07, −0.23) (Figure 3). However, in relation to the sensitivity analysis, there were no significant differences (*p* > 0.05) between the two groups with the removal of Pohl et al. [29,30] studies.

**Figure 4:**
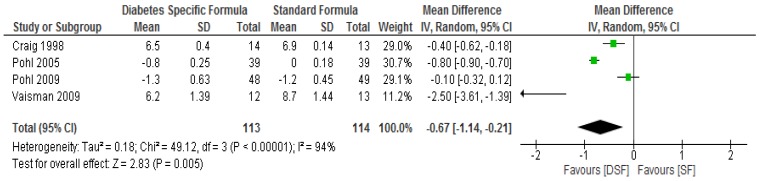
The effect of DSF on Glycated Haemoglobin %.

The glycated haemoglobin was significantly lower (*p* = 0.005) in the DSF group compared to the SF group following meta-analysis (Figure 4) and sensitivity analysis.

### 3.3. The Effect of DSF on Lipid Profile

Based on the systematic review, the outcomes of the studies selected to evaluate the effect of DSF on lipid profile were variable. Craig et al. [31] did not find significant differences with respect to LDL cholesterol and triglyceride between the DSF and the SF groups, but differences were significantly higher (*p* < 0.05) in the DSF group in relation to HDL cholesterol. In two other studies [33,35], the level of HDL cholesterol was significantly higher (*p* < 0.05) in the DSF group compared with the SF group after intervention, but differences were not significant (*p* > 0.05) in relation to triglycerides, total cholesterol and LDL cholesterols. Differences between DSF and SF were also not significant (*p* > 0.05) in terms of triglyceride, total cholesterol, HDL cholesterol and LDL cholesterol in other studies [30,32,38]. In contrast, Pohl et al. [29] reported that there was a significant difference (*p* < 0.05) between the DSF group and the SF group with respect to triglycerides, but differences were not significant (*p* > 0.05) in relation to total cholesterol, HDL and LDL cholesterol. Other studies [36,40] have also shown that DSF is effective in controlling plasma triglyceride.

**Figure 5:**
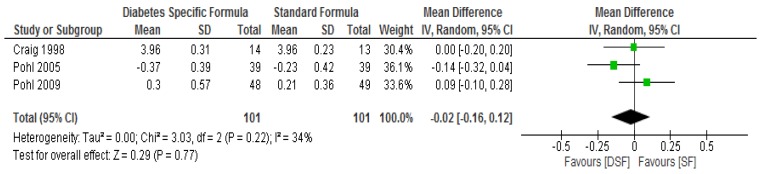
The effect of DSF on total cholesterol (mmol/L).

**Figure 6:**
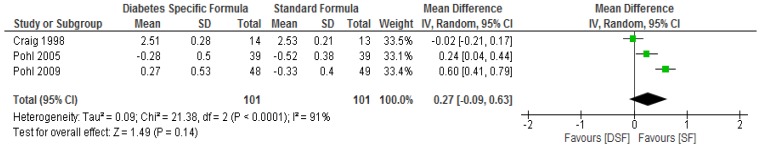
The effect of DSF on LDL cholesterol (mmol/L).

**Figure 7:**
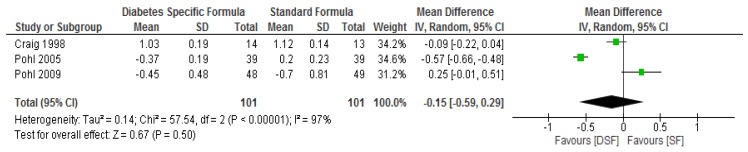
The effect of DSF on Triglycerides (mmol/L).

**Figure 8:**
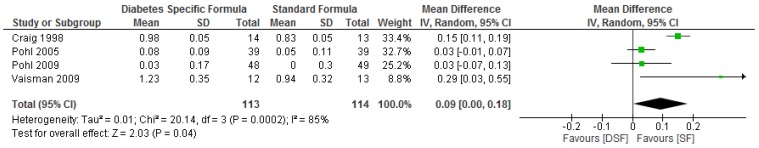
The effect of DSF on HDL cholesterol (mmol/L).

Following meta-analysis, no significant differences (*p* > 0.05) were found between the DSF and SF groups with respect to total cholesterol, LDL cholesterol and triglyceride (Figure 5, Figure 6 and Figure 7). However, the DSF group had a significantly higher level (*p* = 0.04) of HDL cholesterol compared to the SF group after the intervention, with a mean difference of 0.09 (95% CI, 0.00, 0.18) (Figure 8). The results of the sensitivity test for HDL cholesterol demonstrated no significant differences (*p* > 0.05) between the two groups when the Craig et al. [31] and Vaisman et al. [33] studies were removed from the analysis. In addition, the sensitivity analysis showed no significant differences (*p* > 0.05) between the two groups with respect to total cholesterol and triglyceride, while significant differences (<0.05) were observed in relation to LDL cholesterol when the Craig et al. [31] study was removed.

## 4. Discussion

The findings of the systematic review and meta-analysis revealed that DSF was effective in lowering blood glucose (fasting blood glucose and glycated haemoglobin) compared with SF in patients with type 2 diabetes. However, the sensitivity analysis for the fasting blood glucose did not demonstrate a significant difference (*p* > 0.05) with the removal of the Pohl et al. [29,30] studies. In addition, there were no significant differences (*p* > 0.05) between the DSF and SF groups with respect to total cholesterol, LDL cholesterol and triglyceride (although a few studies reported significant differences with respect to triglyceride). Differences in the outcomes of studies in the systematic review were observed with respect to the effect of DSF on HDL cholesterol and the meta-analysis also showed significantly higher levels for the DSF group compared with the SF group. The high level of heterogeneity in the studies included in the meta-analyses may explain why the results of the meta-analysis and the sensitivity analysis were not consistent with respect to HDL cholesterol and fasting blood glucose.

The presence of low glycaemic index (GI) carbohydrate in the form of isomaltulose, the lower amount of carbohydrate and the higher protein content in the DSF may have contributed to the findings of this review [37]. In addition, the presence of mono-unsaturated fatty acids (MUFA) and the different amounts and types of fibre in the DSF compared with SF may be responsible for the observed differences in the fasting blood glucose, glycated haemoglobin and lipid profiles in both groups [18,41]. DSFs are usually higher in fat (40–50% of energy with a significant portion of MUFA) and have a lower carbohydrate level (30–40% of energy) and about 15% of energy is derived from fructose and soluble fibre [20]. DSFs contain carbohydrates with low GI such as non-hydrolysed starches, disaccharides, fibre and fructose in varying amounts which are aimed at controlling postprandial glucose [17,18,19,20]. In contrast, SFs are high in carbohydrate (about 50%) and have low–moderate levels of lipids (about 30%) and do not contain dietetic fibre [17]. A study by Hofman et al. [42] demonstrated that, in 12 enteral formulas examined, the GI ranged from 12 for DSFs up to 61 for SFs. The GI of food is a measure of how quickly the food is digested and the glucose reaches the blood stream [22,43]. Foods with high GI rapidly increase blood glucose and insulin responses after consumption [43,44]. The results from meta-analysis showed that the intake of a low GI diet was associated with reductions in blood glucose parameters [35,43]. In addition, high soluble fibre-containing foods can improve glycaemic control partly due to delayed absorption [36].

Therefore, DSFs may improve glycaemic control through delay in gastric emptying, delayed intestinal absorption of carbohydrate and lower glycaemic response [20]. In the study by Alish et al. [34], the blend of DSF was made up of low glycaemic and slowly digestible carbohydrates, resistant maltodextrin, isomaltulose, sucromalt and prebiotic fibres, including fructo-oligosaccharides. These constituents collectively produce a slow and consistent release of glucose into the blood stream [34]. Isomaltulose is a naturally occurring low GI slowly digestible carbohydrate [18]. The slower hydrolysation of isomaltulose during digestion may be responsible for the slower rise in blood glucose in patients with diabetes on DSF [18]. In addition, the higher protein content of the DSF may have contributed to the lowering of blood glucose parameters by delaying gastric emptying [18].

The use of high fat content, including MUFA, in the DSF may slow the transit time in the gastrointestinal tract and slow the absorption of sugars which could help improve glycaemic control [35,41]. Diets that are high in MUFA have been shown to increase HDL cholesterol and reduce other components of the lipid profiles [35,41]. HDL cholesterol is useful for reducing the risk of cardiovascular disease [35]. The result of the meta-analysis of the current review confirmed the positive role of DSF in increasing HDL cholesterol. However, the sensitivity tests did not demonstrate consistency in terms of the effect of DSF on HDL cholesterol, which could explain why researchers may be reluctant to recommend the use of high fat content in DSF due to the risk of alterations in lipid profiles [41]. This may also be due to the fact that there have been differences in the outcomes of studies on the effect of DSF on lipid profile [41].

## 5. Limitation

The limitation of this review was that only five studies were included in the meta-analysis. In particular, there were fewer studies included for lipid outcomes (three for several parameters) and there was substantial variability in the studies. Therefore, the differences between the meta-analysis and the sensitivity analysis in some of the parameters suggest that those results were not quite consistent, which may be due to the high level of heterogeneity of the studies. Therefore, more studies are needed to address this problem.

## 6. Conclusions

The results provide evidence to suggest that DSF is effective in controlling fasting blood glucose and glycated haemoglobin. In addition, DSF was effective in increasing HDL cholesterol but had no significant effect on other lipid parameters. However, our confidence in these findings would be increased by additional data from further studies. Additional research would also provide the opportunity to refine our understanding of the effect of DSF on cardiometabolic parameters.

## Figures and Tables

**Table 1:** Criteria for considering studies for the review based on the Population, Intervention, Comparator, Outcomes and Study designs (PICOS) Structure.

Inclusion Criteria		Exclusion Criteria
**Population**	Patients with type 2 diabetes and on enteral nutrition irrespective of type of feeding tube.	Patients with type 1 diabetes.
		Pregnant women with gestational diabetes.
		Healthy individuals without diabetes on enteral nutrition.
		Patients with diabetes on parenteral nutrition and parenteral plus enteral nutrition.
		Studies involving animals
**Intervention**	Diabetes specific formulas	Parenteral nutrition, parenteral plus enteral nutrition.
	(Oral nutrition supplement or enteral tube feeding)	
**Comparator**	Standard formulas (Oral nutrition supplement or enteral tube feeding)	Parenteral nutrition and parenteral plus enteral nutrition.
**Outcomes**	Cardiometabolic parameters	Qualitative outcomes such as patient feelings.
**Study Design:**	Randomised Controlled Trials	Letters, comments, reviews, qualitative studies

**Table 2:** Search method for identification of studies.

Patient/Population	Intervention	Comparator	Study Designs	Combining Search Terms	
Patients with type 2 diabetes	Diabetes specific formulas	Standard formulas	Randomised Controlled Trial		
Type 2 diabetes OR type 2 diabetes mellitus OR Diabetes complications OR diabetes mellitus, type 2	Diabetes specific formula OR Diabetes specific form* OR Enteral nutrition OR Enteral* OR Enteral feed OR Enteral feed* OR Enteral form* OR Diabetes formula OR tube feeding OR enteral feeding		Randomised Controlled Trial OR Randomized Controlled Trial OR Randomized Controlled study OR RCT OR Randomized* OR controlled clinical trial OR placebo OR randomly OR trial OR groups	Column 1 AND Column 2 AND Column 3	

**Table 3:** Characteristics of the articles included in this review (*n* = 14).

Citation	Country	Length of Study	Study Type/Design	Sample Size/Description	Age (Years)	Type of Enteral Formula/Feeding Method	Duration of Diabetes (Years)	Study Results/Conclusion
Ceriello et al. [18]	Netherlands	24 h	Randomized, controlled, double-blind, cross-over	*n* = 11	Mean ± SEM	The DSF had 1 kcal/mL and low GI and/or slowly digestible CHO. The SF was isocaloric fibre containing formula.	Mean ± SEM	Administration of DSF lowered glucose profiles.
					67.2 ± 1.3	Bolus Feeding	6.6 ± 1.4 years	Using DSF resulted in significantly lower 24 h and postprandial glucose profiles than fibre-containing SF after bolus administration.
Buranapin et al. [20]	Thailand	180 min	Single centre, prospective, randomized, double blind, cross-over study.	*n* = 30	Mean ± SD	55% CHO, 15% protein, 30% fat for DSF and SF. However, DSF substituted sucrose for combination of fructose, polydextrose and FOS. Bolus Feeding	More than 6 months.	DSF resulted in significantly lower postprandial blood glucose concentration than SF.
			Administration of oral DSF and SF		60.93 ± 11.71			
Pohl et al. [29]	Germany	12 weeks	Randomized, double-blind, controlled, multi-centre trial.	*n* = 78	Median (Range)	DSF contained 37% energy as CHO, 45% as fat, 18% as protein, SF contained 52% energy as CHO, 30% of energy as total fat and 18% as protein.	No data	DSF formula resulted in a more effective glycaemia control than SF, and was comparable in safety.
					Test group (DSF): 71 (42–86)	Continuous Feeding.		DSF significantly decreased triglycerides compared with SF, but differences were not significant in relation to total cholesterol, HDL and LDL cholesterols.
					Control group (SF): 72 (51–87)			
Pohl et al. [30]	Germany	84 days	Parallel design.	*n* = 97	Median (Range)	DSF contained 37% energy as CHO, 45% as fat, 18% as protein, SF contained 52% energy as CHO, 30% of energy as total fat and 18% as protein.	No data	Compared to SF, DSF significantly lowered FBG and improved glycaemic control.
			Stage two of a randomized, prospective, double-blind, controlled, multicentre, parallel group study		DSF: 74 (44–91)	Continuous Feeding.		There were no significant differences between the two groups with respect to TG, TC, HDL and LDL cholesterols.
					SF: 69 (53–86)			
Craig et al. [31]	USA–New York State	3 months	Randomized, double-blind, controlled, parallel group 3 months pilot trial.	*n* = 34	DSF: 82 ± 3 (range 52–94)	Per 1000 mL, DSF contained 1000 kcal, 41.8 g protein, 93. 7 g CHO, 55.7 g fat. SF contained 1060 kcal, 44.4 g protein, 151.7 CHO, 35.9 g fat. Continuous or intermittent feeding.	No data	DSF resulted in lower fasting serum glucose and HbA1c than SF.
					80 ± 2 (range-SF: 52–100)			No significant differences between the DSF and SF groups with respect to LDL cholesterol and TG 3 months post intervention, but the DSF group had significantly higher level of HDL cholesterol than the SF group.
Lansink et al. [32]	Netherlands	4 weeks	Randomized, controlled, double-blind, parallel-group study.	*n* = 44	Mean ± SD	DSF contained 1 kcal/mL, 47 Energy% CHO, 19 Energy% protein, 34 Energy% fat and 2 g fibres/100 mL. The SF contained 50 Energy% CHO, 16 Energy% protein, 34 Energy% fat and 1.5 g fibres/100 mL.	Mean (Range) DSF: 84 (18–216) months	DSF significantly lowered postprandial glucose compared with SF.
					DSF: 65.2 ± 7.4 SF: 64.2 ± 5.9	Bolus Feeding	SF: 66 (10–504) months	Levels of TG, TC, HDL and LDL cholesterols were not significantly different between the two groups at baseline and 4 weeks post intervention.
Vaisman et al. [33]	No data	12 weeks	Randomized, controlled, double-blind, parallel group study.	*n* = 25	Total: 76.2 ± 12.8 years	DSF contained 100 kcal, 45 Energy% CHO, 38 Energy% fat, 17 Energy% protein and 1.5 g/100 kcal fibre. SF contained 100 kcal, 55 Energy% CHO, 30 Energy% fat, 15 Energy% protein, 2 g/100 kcal fibre.	Mean ± SD	The DSF significantly reduced HbA1c compared to SF. No significant effect was found with respect to fasting blood glucose.
					DSF: 73.0 ± 14.7	Bolus, Continuous or intermittent feeding.	Total: 8.6 ± 7.6 years DSF: 5.0 ± 4.9	DSF significantly increased HDL cholesterol, but differences were not significant in relation to TG, TC and LDL cholesterol compared with SF.
					SF: 79.2 ± 10.4			
					SF: 12.6 ± 8.4			
Alish et al. [34]	USA	10 days	Randomized, double blind, two treatment, crossover design.	*n* = 12	Mean ± SEM	DSF had 1.2 kcal/mL, 114.5 g CHO, 17 g/L fibre, 60 g/L protein, 60 g/L fat. SF had 1.2 kcal/mL, 169.4 g CHO, 18 g/L fibre, 55.5 g/L protein, 39.3 g/L fat.	NS	Use of DSF produced lower postprandial glycaemic and insulinemic responses, reduced glycaemic variability, and resulted in less hyperglycaemia, reduced short acting insulin requirements.
			DSF (Postprandial response protocol) vs. SF (Continuous glucose monitoring).		Postprandial: 63.1 ± 1.9	Continuous Feeding.		
					Continuous feed: 74.1 ± 4.0			
Gulati et al. [35]	India	8 months	Open-label, randomized, crossover, pilot single centre study.	*n* = 40	35–60 years	DSF administered was 55 g in 210 mL of water to make 250 mL at standard reconstitution (1 kcal/mL) which can be used as tube feed or oral nutrition supplement. The SF was isocaloric Meal.	No data	DSF demonstrated lower blood glucose and insulin post meal levels than SF.
						Bolus Feeding		The level of HDL cholesterol was significantly higher in the DSF group compared with the SF group after intervention, but differences were not significant in relation to TG, TC and LDL cholesterol.
Hofman et al. [36]	Netherlands	360 min	Randomized, double blind, cross over study involving SF (A), DSF with moderate amount of carbohydrate and MUFA (B) and Test feed with low amount of carbohydrate and high amount of fat (C)	*n* = 12	63 ± 9.4 years	DSF (45 Energy% CHO, 26 Energy% MUFA), SF (49 Energy% CHO, 21 Energy% MUFA). Continuous Feeding.	No data	DSF showed significantly lower glucose levels compared with SF.
								With respect to TG level, the DSF B with a lower amount of fat showed significantly lower levels than test feed C.
Lansink et al. [37]	Netherlands	8 h	Randomized, controlled, double-blind cross-over study	*n* = 24	Mean ± SD	The DSF had 1.5 kcal/mL, high protein, a mixture of 6 different dietary fibre and low GI CHO. SF was isocaloric fibre containing formula.	Median (Minimum and Maximum)	Administration of a new, high-protein DSF during 4 h of continuous feeding resulted in lower glucose and insulin levels compared with a fiber-containing SF. DSF may contribute to lower glucose levels in these patients.
					64.6 ± 10.7	Continuous Feeding.	76.5 months (13, 303)	
Mesejo et al. [38]	Spain	2 years	Prospective, open-label, randomized study	*n* = 157	Median (Q1–Q3)	Per 100 mL, DSF had 100 kcal, 5.7 g protein, 8.2 g CHO, 4.4 g fat. SF had 100 kcal, 5/7 g protein, 10.93/15.3 g CHO, 3.79/5.3 g fat.	No data	DSFs lowered insulin requirements, improved glycaemic control and reduced the risk of acquired infections relative to SF.
					New generation DSF: 57 (43–70)	Continuous Feeding.		Plasma levels of cholesterol and TG were similar across the three treatment groups.
					SF: 60 (45–71)			
					Control DSF: 58 (46–68)			
Voss et al. [39]	USA	240 min	Randomized cross over-study	*n* = 48	Mean ± SEM	DSF had 1 kcal/mL, 47.8 g CHO, 7.2 g fibre, 20.9 g protein, 27.2 g fat. SF had 1.06 kcal/mL, 73 g CHO, 7.2 g fibre, 20.9 g protein, 16.4 g fat.		DSF resulted in lower postprandial blood glucose response compared with SF.
			Double-blinded with three-treatments		56 ± 1.4 years	Bolus Feeding		
Vanschoonbeek et al. [40]	Netherlands	10 days	Randomized, double-blind, cross over study.	*n* = 15	Mean ± SEM	Per 100 mL, DSF had 98 kcal, 1.44 g fibre and 5.44/50 (g/energy%) of fat. SF had 100 kcal, 1.4 g of fibre, 3.4/30 (g/energy%) of fat.),	Mean ± SEM	DSF rich in lowly digestible carbohydrate sources can be equally effective in lowering the postprandial blood glucose response as low-carbohydrate, high-fat enteral formulas without elevating the plasma triglyceride response.
					63 ± 1 years	Bolus Feeding	9 ± 2 years	

Abbreviations: NS (Not stated); DSF (Diabetes Specific Formula); CHO (Carbohydrate); FOS (Fructo-oligosaccharide); GI (Glycaemic Index); HbA1c (Glycated haemoglobin); SF (Standard Formula); LDL (low density lipoprotein) Cholesterol; HDL (high density lipoprotein)Cholesterol; MUFA (mono-unsaturated fatty acid); FBG (fasting blood glucose); TC (total cholesterol); TG (triglycerides); T2DM (type 2 diabetes mellitus).

**Table 4:** Blood glucose parameters among individuals with diabetes (Meta-analysis Data Extraction Table).

Study Reference	Interventions	Pre-and Post Intervention	Fasting Blood Glucose mmol/L Mean ± SD/Median (Quartiles)	Glycated Haemoglobin % Mean ± SD/Median (Quartiles)	Total Cholesterol mmol/L Mean ± SD/Median (Quartiles)	LDL Cholesterol mmol/L Mean ± SD/Median (Quartiles)	HDL Cholesterol mmol/L Mean ± SD/Median (Quartiles)	Triglycerides mmol/L Mean ± SD/Median (Quartiles)
Pohl. et al. [29]	DSF, *n* = 39	Change from baseline	** ∆−1.59 (−3.38 to −0.06)	** ∆−0.8 (−1.5 to −0.5)	** ∆−0.37 (−1.00 to 0.56)	** ∆−0.28 (−1.46 to 0.53)	** ∆0.08 (−0.06 to 0.28)	** ∆−0.37 (−0.36 to 0.38)
	SF, *n* = 39	Change from baseline	** ∆−0.08 (−1.34 to 0.79)	** ∆0.0 (−0.4 to 0.3)	** ∆−0.23 (−1.22 to 0.46)	** ∆−0.52 (−1.48 to 0.04)	** ∆0.05 (−0.10 to 0.32)	** ∆0.203 (−0.07 to 0.84)
Pohl et al. [30]	DSF, *n* = 48	Change from baseline	** ∆−2.17 (−2.55/−1.33)	** ∆−1.30 (−2.60/−0.10)	** ∆0.30 (−1.22/1.06)	** ∆0.27 (−0.71/1.40	** ∆0.03 (−0.26/0.4)	** ∆−0.45 (−1.65/0.27)
	SF, *n* = 49	Change from baseline	** ∆−0.67 (−0.90/−0.10)	** ∆−1.20 (−2.35/−0.55)	** ∆0.21 (−1.02/0.48)	** ∆−0.33 (−1.03/0.56)	** ∆0.00 (−0.22/0.28)	** ∆−0.70 (−1.50/1.73)
Craig et al. [31]	DSF, *n* = 14	Baseline	* 7.3 ± 0.4	* 6.9 ± 0.3	* 4.16 ± 0.31	* 2.66 ± 0.23	* 1.01 ± 0.05	* 0.97 ± 0.13
		Final	6.7 ± 0.7	6.5 ± 0.4	3.96 ± 0.31	2.51 ± 0.28	0.98 ± 0.05	0.91 ± 0.17g/L
	SF, *n* = 13	Baseline	* 6.9 ± 0.6	* 6.9 ± 0.5	* 4.21 ± 0.18	* 2.69 ± 0.15	* 0.98 ± 0.05	* 0.9 ± 0.07
		Final	8.3 ± 1.7	6.9 ± 0.14	3.96 ± 0.23	2.53 ± 0.21	0.83 ± 0.05	1.06 ± 0.12 g/L
Lansink et al. [32]	DSF, *n* = 21	Baseline	* 8.32 ± 0.33	No data	No data	No data	No data	No data
		Final	8.13 ± 0.33					
	SF, *n* = 22	Baseline	* 7.73 ± 0.22	No data	No data	No data	No data	No data
		Final	8.22 ± 0.26					
Vaisman et al. [33]	DSF, *n* = 12	Baseline	No data	*** 6.9 ± 0.3	No data	No data	*** 1.04 ± 0.08	No data
		Final		6.2 ± 0.4			1.23 ± 0.10	
	SF, *n* = 13	Baseline	No data	*** 7.9 ± 0.3	No data	No data	*** 1.06 ± 0.08	No data
		Final		8.7 ± 0.4			0.94 ± 0.09	

Abbreviations: NS (Not stated); SD (Standard deviation); SEM (Standard Error of Mean); ∆ (Change from baseline) * Mean ± SD; ** Median (Quartiles); *** Mean ± SEM.
